# Organic Bulk–Heterojunction Blends with Vertical Phase Separation for Enhanced Organic Photodetector Performance

**DOI:** 10.3390/polym16213040

**Published:** 2024-10-29

**Authors:** Chih-Ping Chen, Yan-Cheng Peng, Bing-Huang Jiang, Ming-Wei Hsu, Choon Kit Chan, He-Yun Du, Yang-Yen Yu

**Affiliations:** 1 Department of Materials Engineering, Ming Chi University of Technology, New Taipei City 24301, Taiwan; cpchen@mail.mcut.edu.tw (C.-P.C.); m09188024@mail2.mcut.edu.tw (Y.-C.P.); a0988808118@gmail.com (B.-H.J.); 2 Department of Chemical and Materials Engineering, Chang Gung University, Taoyuan City 33302, Taiwan; 3 Cagu International Co., Ltd., Kaohsiung 80652, Taiwan; mark.h@cagugroup.com; 4 Mechanical Engineering Department, Faculty of Engineering and Quantity Surveying, INTI International University, Nilai 71800, Negeri Sembilan, Malaysia; choonkit.chan@newinti.edu.my; 5 Department of Chemical Engineering, Ming Chi University of Technology, New Taipei City 24301, Taiwan; heyundu@mail.mcut.edu.tw

**Keywords:** organic photodetector, Kelvin probe force microscopy, vertical phase separation, heart rate sensing, ternary component blending technology, ultra-high detectivity

## Abstract

The ternary blending strategy is a fundamental approach that is widely recognized in the field of organic optoelectronics. In our investigation, leveraging the inherent advantages of the ternary component blending methodology, we introduced an innovative design for organic photodetectors (OPDs) aimed at reducing the dark current density (J_d_) under reverse bias. This pioneering effort involved combining two distinct conjugated molecules (IT-4F and IEICO-4F) with a conjugated polymer (PM7), resulting in a composite material characterized by a well-defined vertical phase separation. To thoroughly explore device performance variations, we utilized a comprehensive array of analytical techniques, including atomic force microscopy (AFM) cross-section methodologies and Kelvin probe force microscopy (KPFM). Through the optimization of the blend ratio (PM7:IT-4F: IEICO-4F at 1:0.8:0.2), we achieved significant advancements. The resulting OPD demonstrated an exceptional reduction in JD, reaching a remarkably low value of 4.95 × 10^−10^ A cm^−2^, coupled with an ultra-high detectivity of 4.95 × 10^13^ Jones and an outstanding linear dynamic range exceeding 100 dB at 780 nm under a bias of −1V. Furthermore, the attained cutoff frequency reached an impressive 220 kHz, highlighting substantial improvements in device performance metrics. Of particular significance is the successful translation of this technological breakthrough into real-world applications, such as in heart rate sensing, underscoring its tangible utility and expanding its potential across various fields. This demonstrates its practical relevance and underscores its versatility in diverse settings.

## 1. Introduction

Across various applications, spanning from photosensing to communication, organic photodetectors (OPDs) represent highly promising solutions. Their distinct advantage lies in their cost-effectiveness compared to traditional photodetection technologies, as extensively evidenced in the literature [1,2,3,4,5]. This inherent trait not only facilitates large-scale manufacturing but also makes it economically feasible. Moreover, the inherent flexibility of OPDs extends to their material composition and device architecture, offering a wide range of design possibilities [6]. A diverse array of organic materials can be incorporated in OPDs, providing unmatched versatility in tailoring their optoelectronic properties to suit specific application needs. Additionally, the inherent flexibility of OPDs makes them well suited for applications requiring conformable and adaptable photodetection devices, thus making them and their portability indispensable in fields such as artificial intelligence, automotive technologies, and the burgeoning Internet of Things [5,7,8,9]. Collectively, these attributes highlight the compelling value of OPDs across various industries, paving the way for innovative and cost-efficient solutions in the realms of photosensing and communication.

In the quest to enhance OPD performance, a significant amount of research has been dedicated to achieving a heightened photoresponse/detectivity (D*) [1,2,10], expanding linear dynamic ranges (LDRs) [11], and minimizing response times [12], all of which are crucial parameters for optimizing device functionality. Particularly noteworthy is the intricate interplay between device operation and performance, where, under reverse bias conditions, the dark current magnitude and the mechanisms governing charge injection/bulk thermal generation play pivotal roles in shaping the value of D* in OPDs [13,14]. This underscores the nuanced nature of device behavior as well as the need for a comprehensive understanding to drive performance enhancements. Of significant interest are photodiode-based OPDs, which share structural similarities with organic photovoltaics, presenting a promising avenue for the development of self-powered photodetectors [15,16]. This heralds a paradigm shifts toward sustainable and energy-efficient photodetection solutions, aligning with the broader global initiatives toward sustainability and the adoption of green technology.

OPDs have emerged as a technology of interest across a diverse spectrum of fields, encompassing applications ranging from advanced camera systems, automotive sensing lenses, and rangefinders to the burgeoning domain of heart rate monitors, among others [1,2,5,17]. However, the unprecedented global health crisis precipitated by the outbreak of COVID-19 has underscored the critical imperative for bolstered medical resources and innovative technological solutions. Historically, due to their unparalleled precision and exceptionally low noise characteristics, silicon photodetectors have held sway in medical instrumentation. Nevertheless, OPDs offer a distinctive array of advantages that render them uniquely poised for integration within medical applications. By judiciously selecting near-infrared (NIR) absorbers, OPDs exhibit remarkable capabilities in detecting NIR radiation, meaning they are able to glean invaluable physiological insights that are essential for medical diagnoses and continuous monitoring [18,19,20]. It is evident that the distinctive attributes inherent to OPDs position them as potential game-changers within the medical landscape; their potential to engender a paradigm shift in healthcare practices heralds a future characterized by a heightened efficacy and precision in medical diagnostics and patient care protocols.

OPDs encounter formidable challenges that impede their evolution, including the persistent issue of a high dark current density (J_D_) and the notable gap in comprehension regarding the intricate interplay between device performance and film morphology [21]. In our investigation, we embarked on an exploration of NIR OPDs with photodiode configurations, meticulously crafting a ternary blend that includes a conjugated polymer donor (D) and two small-molecule acceptors (A). This deliberate formulation was meticulously engineered not only to achieve heightened response speeds but also to mitigate the dark current density (J_D_), thus enhancing the effectiveness of OPDs in photosensing applications. Our investigation also delves into the vertical phase separation dynamics of OPDs, elucidating their profound implications on J_D_, as examined in previous studies [22,23]. Specifically, we meticulously explored the vertical phase separation phenomena within the poly(2,6-(4,8-bis(5-(2-ethylhexyl-3-chloro)thiophen-2-yl)-benzo [1,2-b:4,5-b’]dithio phene))-alt-(5,5-(1′,3′-di-2-thienyl-5′,7′-bis(2-ethylhexyl)benzo [1′,2′-c:4′,5′-c’] dithiophene-4,8-dione) (PM7): 3,9-bis(2-methylene-((3-(1,1-dicyanomethylene)-6,7-difluoro)-indanone))-5,5,11,11-tetrakis(4-hexylphenyl)-dithieno [2,3-d:2′,3′-d’]-s-indaceno [1,2-b:5,6-b’]dithiophene (IT-4F) blend, employing cutting-edge cross-section atomic force microscopy (AFM) and Kelvin probe force microscopy (KPFM) to unravel the intricate nanostructural nuances underpinning device performance enhancements.

Furthermore, we endeavored to enhance the NIR responsiveness of OPDs by strategically incorporating a tertiary component material: 2,2’-((2Z,2’Z)-(((4,4,9,9-tetrakis(4-hexylphenyl)-4,9-dihydro-sindaceno [1,2-b:5,6-b’]dithiophene-2,7-diyl)bis(4-((2-ethylhexyl)oxy)thiophene-5,2-diyl))bis(methanylylidene))bis(5,6-difluoro-3-oxo-2,3-dihydro -1H-indene-2,1-diylidene)) dimalononitrile (IEICO-4F). Notably, our experimental results culminated in achieving outstanding OPD performance metrics, which are characterized by an ultra-low dark current density of 4.95 × 10^−10^ A cm^−2^ at a bias of –1 V, along with an unparalleled detection ratio, reaching an impressive 4.95 × 10^13^ Jones. Additionally, aiming to enhance the portability and versatility of heart-rate-monitoring devices, we succeeded in significantly elevating the cutoff frequency to a notable 220 kHz, highlighting its high applicability across various operational scenarios.

Looking forward, we envisage seamlessly integrating this transformative technology into blood oxygen detection, promising enhanced diagnostic capabilities for healthcare professionals and heralding a new frontier in medical diagnostics and patient care.

## 2. Experimental Section

The process for constructing OPD devices adhered to a meticulously planned procedure following an indium tin oxide (ITO)/ZnO/active layer/MoO_3_/Ag configuration, which was carefully engineered to maximize device performance. To prepare an effective base for the devices, the ITO glass substrate, procured from Sanyo, Japan, with a resistivity of 6.4 Ω square^−1^, underwent a comprehensive series of preparatory steps. Initially, the ITO glass was meticulously patterned using precision etching techniques, which was followed by a rigorous cleaning regimen involving detergent; sequential ultrasonic cleaning with water, acetone, and isopropyl alcohol (IPA); and culminating in thorough drying at 120 °C for 30 min to ensure pristine substrate conditions. The formulation of the sol–gel ZnO precursor solution constituted a critical phase during which zinc acetate (3.15 g), ethanolamine (0.9 mL), and 2-methoxyethanol (29.1 mL) were combined and stirred for 3 days to achieve optimal homogeneity and reactivity. The sol–gel ZnO precursor solution was filtered through a fine 0.45 µm PTFE filter to eliminate impurities and was then carefully applied onto the ITO glass substrate via spin coating at 3000 rpm for 30 s. This was followed by a meticulously controlled reaction at 170 °C for 20 min under ambient conditions to promote the conversion into a durable ZnO layer. After the deposition of ZnO, the treated ITO glass substrate underwent additional treatment, involving exposure to oxygen plasma for 5 min, to enhance the surface properties favorable for subsequent layer deposition.

For the fabrication of the active layer, a solution consisting of PM7:IT-4F: IEICO-4F at varying weight ratios (1:0.8:0, 1:0.8:0.2, and 1:0.8:0.4) was formulated at a concentration of 10 mg/mL in a solvent blend of chlorobenzene/dichlorobenzene supplemented with 3% diiodooctane, guaranteeing optimal dispersion and compatibility. This precursor solution underwent extended stirring overnight to achieve maximum homogeneity and dissolution. Following this, both the blend solution and the preheated ITO/ZnO substrate were maintained at 90 °C to ensure ideal deposition conditions. The solution, held at the required temperature, was carefully coated onto the substrate using a spin-coating technique, where the thickness of the active layer was precisely controlled by adjusting both the solution temperature and the spin-coating rate (from 800 to 2000 rpm) for precise thickness adjustments (from 200 to 400 nm). The subsequent deposition of the MoO_3_ (thickness: 3 nm) and Ag (thickness: 100 nm) layers atop the active layer followed a meticulously planned process, whereby a precisely engineered mask facilitated the deposition of these layers via evaporation equipment operated at a pressure of less than 10^−6^ torr, ensuring unparalleled control and precision.

## 3. Measurements

The thickness of each layer was measured using either a sophisticated surface profiler (Laboratory, E200, Kosaka Laboratory Ltd, Sotokanda Chiyoda-ku, Tokyo, Japan) or an atomic force microscope (Bruker Dimension Edge, BRUKER, Billerica, MA, USA), which was operated carefully in tapping mode. Furthermore, the atomic force microscope (Bruker Dimension Edge, BRUKER, Billerica, MA, USA) served a dual purpose, assessing the thickness but also comprehensively collecting topographical and phase images of the active layers manufactured under an array of experimental conditions. Additionally, optical microscopy (OM) facilitated the observation of surface morphologies at larger scales, providing a more holistic understanding of the fabricated layers. Moreover, the dark current density of the OPDs was evaluated under both dark and ambient conditions, maintained at approximately room temperature (26 °C) with a humidity level of about 50%, utilizing a programmable power controller (Keithley model 2636A, Tektronix, Beaverton, OR, USA) for precise measurements. An external quantum efficiency (EQE) response measurement system (QE-R3011, Enlitech, Kaohsiung City, Taiwan) was used to capture the EQE spectra and responsivities of the OPDs. This involved meticulously calibrating monochromatic light beams from a commercial light source (Newport, TLS-300XR, Newport, CA, USA) using a Si photodetector (Newport, 818-UV, Newport, CA, USA) and modulating at a frequency of 250 Hz through an optical chopper system before illuminating the devices; the ensuing responses were meticulously recorded using a lock-in amplifier (Signal Recovery 7225, Ametek, PA, USA). To assess the LDRs, the power density of the LED light source (Thorlabs, M530L4/M780L3, Thorlabs, Newton, NJ, USA) underwent meticulous correction utilizing a Newport UV 818-L instrument before passing through a motorized filter wheel (Thorlabs, FW102CNEB, Thorlabs, Newton, NJ, USA) to ensure uniform irradiation across the OPD device area. Additionally, for evaluating the frequency response, a light beam was generated from a commercial LED (Thorlabs, Newton, NJ, USA) emission wavelength: 530/780 nm) with a flux density of 1 mW cm ^2^, which was followed by precisely triggering the OPD device response using a function generator (Tektronix, AFG3102C, Tektronix, Beaverton, OR, USA). Subsequently, a low-noise current preamplifier, featuring an A/V gain factor of 105 and devoid of any bandwidth filter (Ametek, model 5182, Ametek, Inc., Berwyn, PA, USA), was utilized to accurately convert the signal response from photocurrent to photovoltage, which was then displayed and recorded using a 2.5 GHz oscilloscope (Teledyne LeCroy, Wave Runner 625Zi, Teledyne LeCroy, NY, USA).

## 4. Results and Discussion

**Figure 1:**
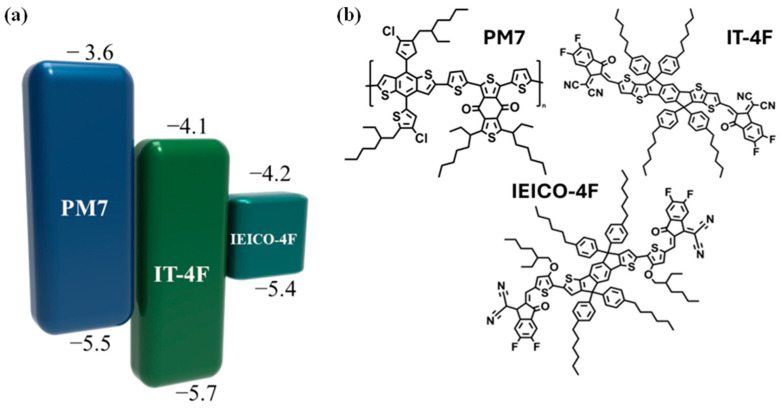
(**a**) Energy levels and (**b**) chemical structures of materials.

Figure 1 shows a visual representation of the diverse array of materials meticulously curated in this comprehensive study. Notably, the inclusion of chlorine or fluorine atoms within the molecular structure imparted a pronounced dipole moment, engendering heightened intermolecular interactions, as visually depicted in Figure 1a. Specifically, the organic semiconductor PM7, characterized by its deeply positioned highest occupied molecular orbital (HOMO) energy level [24], was judiciously selected to serve as the electron donor, forging a symbiotic relationship with the acceptor molecule, IT-4F [24,25]. This strategic pairing was predicated upon IT-4F’s remarkable photostability and the concomitant reduction in the lowest unoccupied molecular orbital (LUMO) energy level facilitated by fluorination [26], which is a pivotal factor contributing to the effective mitigation of dark current under reverse bias conditions, as substantiated by prior investigations. In a bid to extend the spectral responsiveness of the fabricated devices toward the NIR regime, a critical prerequisite for enabling heart-rate-sensing capabilities, we introduced IEICO-4F as the third component, which was characterized by its low bandgap properties [27,28]. The devices were meticulously fabricated by employing a precise layering configuration consisting of ITO as the substrate, which was followed by a ZnO layer; the active layer comprised a blend of PM7, IT-4F, and IEICO-4F; a MoO3 layer; and finally, a top layer of silver. The amount of IEICO-4F, a crucial component, was systematically varied within the PM7:IT-4F:IEICO-4F blend, specifically at weight ratios of 1:0.8:0.2 and 1:0.8:0.4, which were denoted as IEICO 0.2 and IEICO 0.4, respectively. Additionally, binary devices devoid of IEICO-4F were fabricated as the control group to enable rigorous comparative analysis. To ensure consistency and reliability in our experimental approach, meticulous optimization procedures were employed to achieve uniformity in film thickness across all the fabricated devices prior to subsequent measurements. This meticulous approach to fabrication and optimization was essential in ensuring the integrity and reproducibility of our experimental data sets, enhancing the robustness and reliability of our findings.

**Figure 2:**
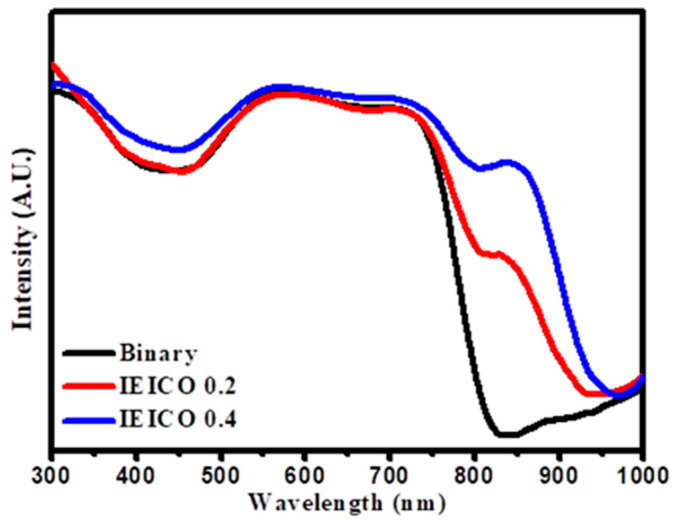
The UV–Vis spectra of each absorbance layer.

Figure 2 shows the absorption spectra exhibited by the film blends under investigation. Notably, our observations unveiled a discernible augmentation in NIR absorption capabilities, concomitant with the incorporation of IEICO-4F within the ternary blend composition, as visually depicted in Figure 2 This noteworthy enhancement in NIR absorption underscores the pivotal role played by IEICO-4F in extending the spectral responsiveness of the fabricated OPDs toward the NIR regime, facilitating the realization of enhanced device performance metrics.

**Figure 3:**
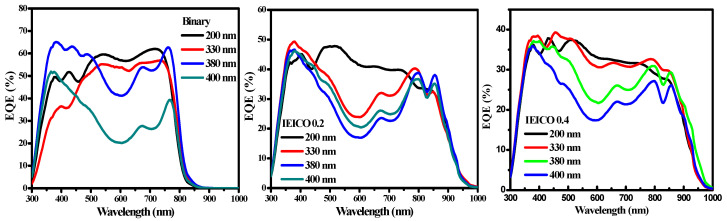
The EQE spectra of devices with different absorbance layer film thicknesses.

In our endeavor to unravel the intricate nuances governing OPD performance, we meticulously scrutinized the dark current density (J_D_) values corresponding to each fabricated OPD variant. As delineated in Figure 3, we embarked on a comprehensive examination of the EQE responses exhibited by OPDs featuring varying active layer thicknesses. Intriguingly, our empirical findings revealed a prominent trend whereby an increase in active layer thickness, within the prescribed range of 200–400 nm, elicited a corresponding diminution in EQE response. This decrease can be attributed to the constraints on carrier transport within the organic semiconductor layer, as there is a trade-off relationship between J_D_ and the EQE response in OPDs. After meticulous analyses and comparative assessments across the spectrum of investigated thicknesses, we discerned that an active layer thickness of 330 nm was the optimal configuration, affording the most favorable balance between JD mitigation and EQE response enhancement. This culminated in the attainment of optimized OPD performance characteristics. Consequently, the active layer thickness of 330 nm was judiciously earmarked for subsequent in-depth evaluation and further refinement, signifying a critical milestone in our ongoing quest toward enhancing the efficacy and functionality of organic photodetection technologies.

**Figure 4:**
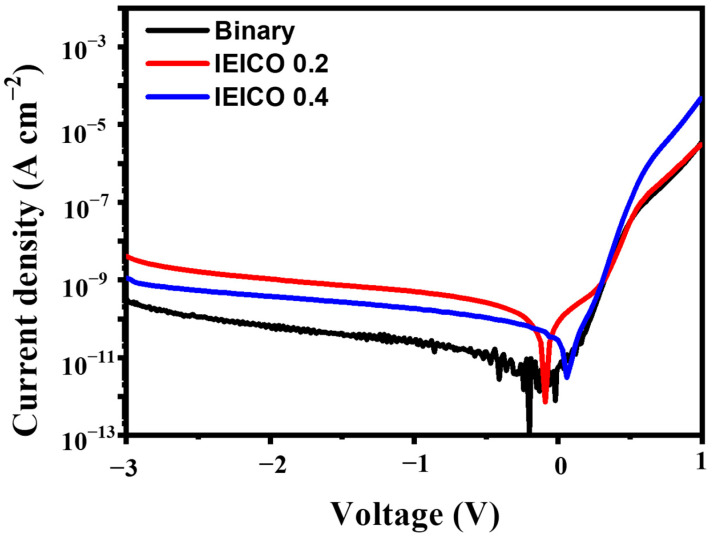
J_D_ of the respective OPDs.

Figure 4 provides a comprehensive depiction of the dark current (J_D_) curves characteristic of the OPDs under scrutiny, unveiling intriguing insights into the impact of IEICO-4F incorporation on J_D_ values. Notably, our findings revealed a marginal uptick in J_D_ levels upon the addition of IEICO-4F, as visually represented in Figure 2. Specifically, under a bias of −1 V, the binary device configuration exhibited an exceedingly low dark current density, registering a mere 2.69 × 10^−11^ Acm^−2^, while the IEICO 0.4 device showcased a modest increase to 1.83 × 10^−10^ A cm^−2^. In stark contrast, the IEICO 0.2 device emerged as the epitome of excellence, manifesting exceptional performance metrics characterized by a dark current density of 4.95 × 10^−10^ A cm^−2^, underscoring the propensity of IEICO-4F incorporation to engender a discernible augmentation in JD levels, which is concomitant with a notable extension of NIR responsiveness.

**Figure 5:**
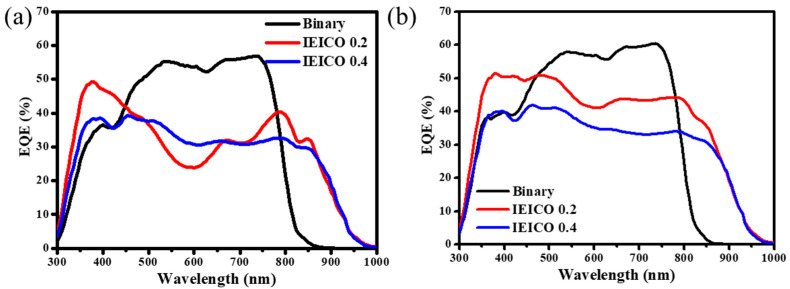
EQE responses of OPDs under (**a**) 0 V and (**b**) –1 V.

Moreover, the accuracy of the observed photoresponse across various wavelengths was validated through meticulous EQE measurements, as detailed in Figure 5 Notably, upon comparative analysis with Figure 5a, it became evident that a pronounced NIR response was evident for the OPDs within the spectral range of 850~950 nm, aligning seamlessly with the absorption characteristics exhibited by IEICO-4F. Notably, the IEICO 0.2 exhibits a remarkable difference in the shape of its EQE curves at 0 V and −1 V. This observation suggests that IEICO-4F, with its deeper LUMO energy level compared to IT-4F, may trap electrons to some extent. However, this trapping effect is likely mitigated by applying an increased bias. This explains why the difference in the shape of EQE curves at 0 V and −1 V was only apparent in the IEICO 0.2 blend. Intriguingly, within the NIR spectrum, an escalation in IEICO-4F content failed to induce a commensurate enhancement in NIR EQE values, suggesting that the optimized blend ratio (IEICO 0.2) established a harmonious balance in carrier transport dynamics within the ternary blend configuration. As highlighted in Figure 5b, a discernible augmentation in EQE response under a −1 V bias emerged, reaffirming the effectiveness of the IEICO 0.2 configuration in delivering superior OPD performance attributes, epitomizing a delicate equilibrium between JD mitigation and spectral responsiveness enhancement.

**Figure 6:**
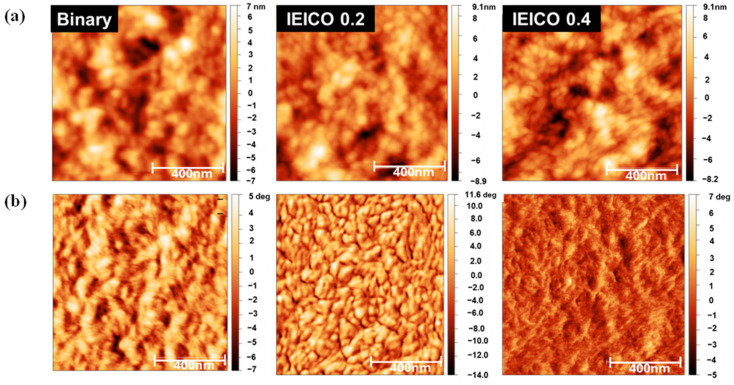
AFM surface images of absorber layer of binary, IEICO 0.2, and IEICO 0.4; (**a**) topographic image and (**b**) phase images.

As depicted in Figure 6, our meticulous examination unveiled a striking semblance in the topographical morphology exhibited across the diverse OPD variants under investigation. Notably, the binary, IEICO 0.2, and IEICO 0.4 configurations manifested distinct roughness values of 2.136, 2.425, and 2.652 nm, respectively, presenting invaluable insights into the nuanced impact of IEICO-4F incorporation on surface morphology characteristics. Intriguingly, a discernible escalation in surface roughness was observed concomitant with the progressive addition of IEICO-4F, heralding a gradual transition from a relatively smooth surface terrain to a markedly roughened morphology. This observation underscores the intricate interplay between material composition and surface morphology in shaping the performance attributes of OPDs.

To investigate the morphological differences between the binary and IEICO-4F-incorporated films, AFM in tapping mode was employed. The AFM images, with a scan area of 1 μm × 1 μm, are presented in Figure 6. Upon meticulous scrutiny of the AFM phase images, a profound dichotomy in morphological attributes emerged between the binary and IEICO-4F-incorporated films. Specifically, the binary films exhibited a homogenously mixed morphology characterized by intimate blending between the polymer and IT-4F components, which is indicative of a well-defined nanophase segregation blend. In stark contrast, the IEICO 0.2 blend showcased a conspicuous phase separation phenomenon, wherein the polymer component assumed a continuous phase configuration while the small molecule counterpart dispersed as discrete domains. This delineation of distinct phases within the IEICO 0.2 blend configuration underpins the observed augmentation in NIR EQE, as the segregated IEICO-4F domains efficaciously capture NIR photons, improving device performance. This intricate understanding of morphological variations sheds light on the underlying mechanisms influencing OPD functionality and paves the way for targeted optimization strategies. Conversely, the IEICO 0.4 blend configuration exhibited a less pronounced phase segregation morphology, characterized by a diminished degree of spatial demarcation between the constituent components, culminating in inefficient charge dissociation dynamics and constricted transport pathways within the device architecture. This inherent limitation, engendered by the heightened presence of IEICO-4F within the blend matrix, impedes the formation of well-defined phases, yielding a suboptimal morphology conducive to device performance optimization. All in all, the incorporation of an appropriate amount of IEICO-4F can enhance the phase separation of the blend, leading to the formation of IEICO-4F-rich domains that contribute to enhanced NIR response. However, an excessive amount of IEICO-4F can hinder the formation of well-defined nanophase segregation in the blend, ultimately leading to a decrease in overall response. This elucidates the rationale behind the observed discrepancy in NIR response between the IEICO 0.2 and IEICO 0.4 OPD configurations, despite the higher blend ratio of IEICO-4F within the latter configuration, underscoring the critical importance of achieving a delicate equilibrium in ternary blend compositions to unlock the full potential of organic photodetection technologies. This intricate understanding highlights the need for finely tuned blend ratios in optimizing the morphology and enhancing the performance of OPDs, advancing the frontier of organic photodetection.

**Figure 7:**
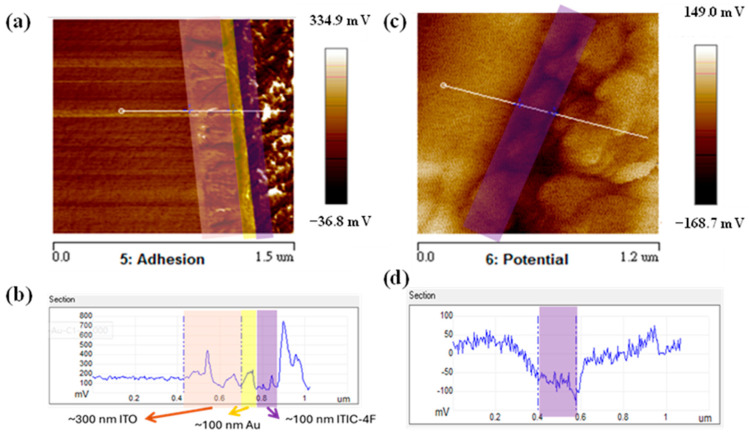
AFM images of (**a**) cross-section adhesion and (**b**) 1D adhesion data; (**c**) KPFM and (**d**) 1D-KPFM data for PM7: IT-4F blend.

Subsequently, we embarked on a comprehensive analysis involving AFM cross-section adhesion measurements of the ITO/Au/absorber layers (binary), as meticulously depicted in Figure 7; in this way, invaluable light is shed on underlying factors contributing to the remarkable properties of PM7:IT-4F-based OPDs. Notably, the delineation of layer thicknesses revealed ITO and active layers measuring 300 nm and 600 nm, respectively, with an intervening Au layer (100 nm) serving as a crucial intermediary. This detailed characterization of layer thicknesses provides crucial insights into the structural integrity and adhesive properties of the device components, which is essential for optimizing device performance and durability. In our endeavor to comprehensively understand the impact of vertical phase separation on device functionality, we conducted KPFM measurements aimed at elucidating the influence of this phenomenon on the built-in potential of the device architecture. Notably, the AFM cross-section adhesion image depicted in Figure 7a vividly elucidates the discernible presence of discrete material domains proximal to the ITO/Au interface, presenting compelling evidence for the phenomenon of vertical phase separation pervading within the blend film matrix. This observation underscores the pivotal role played by segregated small molecules in fostering enhanced charge separation and facilitated transport pathways within the device architecture, fortifying its operational efficacy. This intricate understanding of vertical phase separation provides crucial insights into the underlying mechanisms driving device performance, informing future optimization strategies for organic photodetection technologies. Our thorough analysis further revealed a pronounced dark current suppression in the OPD configuration at 0 V and under reverse bias conditions, which is a phenomenon intricately linked to vertical phase separation, as succinctly delineated by the one-dimensional data depicted in Figure 7b. Furthermore, our KPFM measurements unveiled a conspicuous drop in potential across the device architecture, as vividly depicted in Figure 7c, with the approximately 200 nm thick post-ITO/Au interface region manifesting a discernible potential difference of approximately −130 mV; we quantified this difference, as shown in Figure 7d. This is indicative of the critical role played by the small molecule constituents in shaping device functionality. This comprehensive investigation sheds light on the underlying mechanisms governing charge transport and potential distribution within the OPD structure, offering valuable insights for optimizing device performance and functionality.

To corroborate the veracity of our findings, we meticulously conducted KPFM measurements on pure materials, unequivocally validating the distinctions inherent within the testing function. As shown in Table 1, the work functions of PM7 and IT-4F are −5.3647 eV and −5.6447 eV, respectively, underscoring a discernible difference of approximately −110 meV between two materials that not only affirms the fidelity and reliability of our data but also substantiates the presence of IT-4F beneath the film and corroborates the phenomenon of vertical phase separation. The driving force behind vertical phase separation is typically attributed to the difference in surface energy between the two materials involved [29]. In such a scenario, the material with lower surface energy tends to migrate to the surface of the blend film and vice versa. As reported, PM7 exhibits a lower surface energy compared to IT-4F. Consequently, PM7 tends to migrate to the surface of the blend film, leading to the accumulation of IT-4F at the bottom of the blend film. A well-defined vertical phase separation has been shown to suppress dark current [30]. This is because the acceptor-rich layer at the bottom and the donor-rich layer at the top effectively inhibit charge injection due to the favorable energy level alignment in an inverted device configuration. Armed with these profound insights, we proceeded to comprehensively evaluate the photoelectric performance characteristics of the material under scrutiny, paving the way for a deeper understanding and appreciation of its operational intricacies. This meticulous validation approach enhances the robustness of our conclusions and contributes to a more comprehensive understanding of the material’s behavior in OPD applications.

We calculated device responsivity (*R*) using the following equation:

$$R\left(\lambda \right)=EQE\left(\lambda \right)\times \frac{q\lambda }{h\nu }\left(AW^{-1}\right)$$

where λ is the incident wavelength, *hυ* is the incident photon energy, *h* is Planck’s constant, υ is the frequency, q is the elementary charge, and c is the speed of light.

**Figure 8:**
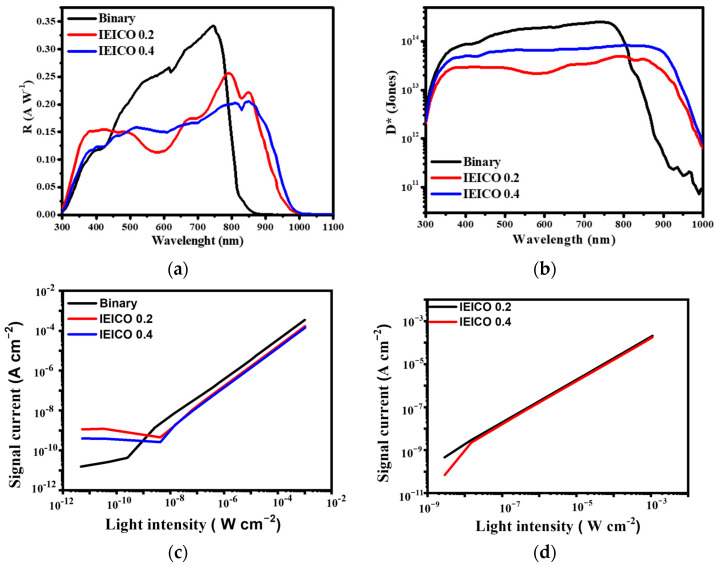
The (**a**) *R*, (**b**) D*, (**c**) LDR (530 nm) and (**d**) LDR (780 nm) of OPDs under –1 V bias.

We determined the detectivity [D* (Jones)] using the following equation [31,32]:

$$D^{*}=\frac{R}{\sqrt{2q\;J_{d}}}$$

where *R* is the responsivity and *q* is the elementary charge (1.6 × 10^−19^ C). We adopt the *J_d_* of the OPDs at different biases and only consider shot noise, ignoring the Johnson noise and flicker noise (1 × *f* ^−1^). As depicted in Figure 8, the R values (at the maximum wavelength) for the binary, IEICO-0.2, and IEICO-0.4 devices were calculated as 0.34, 0.26, and 0.22 AW^−1^ at a bias of −1 V, providing valuable insights into the spectral responsiveness of the fabricated OPDs. Additionally, the corresponding detection ratio [D* (Jones)] values (at −1 V) for the binary, IEICO-0.2, and IEICO-0.4 devices were calculated as 2.53 × 10^14^, 4.95 × 10^13^, and 8.18 × 10^13^ Jones, respectively, highlighting the exemplary performance achieved in this study and significantly surpassing the benchmarks established in numerous recent publications (Table 2). This robust performance underscores the efficacy of the innovative approach employed herein, paving the way for enhanced OPD performance in diverse applications. These findings not only elucidate the superiority of the developed OPD configurations but also offer promising prospects for further advancements in organic photodetection technologies.

**Figure 9:**
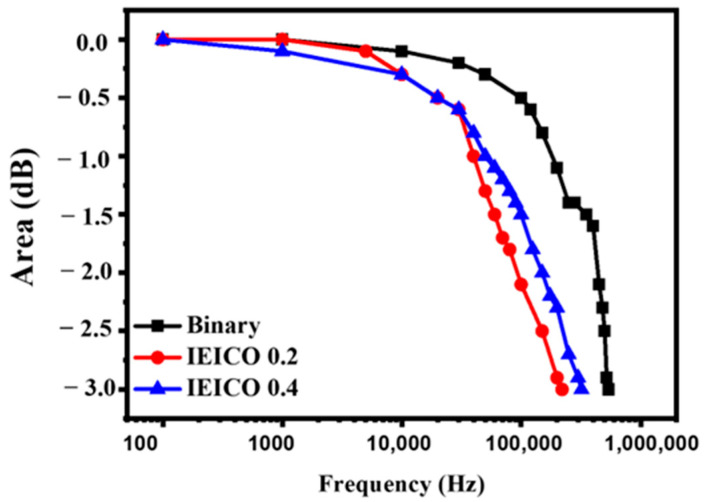
Cutoff frequency of OPDs.

Following preliminary assessments, a comprehensive evaluation of each parameter of the LDR was meticulously conducted to gauge the impact of IEICO-4F incorporation on device performance. As illustrated in Figure 7c,d, the integration of IEICO-4F exerted negligible effects on LDR performance characteristics. The binary device showcased an exceptional performance, achieving an impressive LDR of 108 dB under a 530 nm light source at a bias of −1 V. Similarly, the IEICO 0.2 and IEICO 0.4 devices exhibited remarkable LDR performances with values of 108 dB and 111 dB, respectively, underscoring the robustness and versatility of the fabricated OPDs across diverse operational scenarios, including NIR light sources. Additionally, an assessment of the performance of both devices under a 780 nm NIR light source at −1 V yielded LDR values of 112 dB and 110 dB for IEICO 0.2 and IEICO 0.4, respectively, further attesting to the broad applicability and efficacy of the fabricated OPDs in capturing and processing optical signals across a wide spectral range. Leveraging the exceptional performance attributes of the PM7:IT-4F blend, the strategic introduction of IEICO-4F served to effectively broaden the absorption spectrum, facilitating the development of broadband OPDs capable of catering to a wide range of spectral inputs. Moreover, cutoff frequency measurements played a pivotal role in identifying the operational frequency range (from 0.1 to 1000 kHz) of the devices. As depicted in Figure 9, at a bias of −1 V, the cutoff frequencies for the binary, IEICO 0.2, and IEICO 0.4 devices were determined to be 350 kHz, 220 kHz, and 320 kHz, respectively. These findings unequivocally demonstrate that the addition of IEICO-4F engendered only minimal changes in the cutoff frequency, which attributed to IEICO-4F slightly inhibited the charge carrier mobility [17], affirming the stability and robustness of the fabricated OPDs across diverse operational conditions. This comprehensive analysis provides valuable insights into the optimization of OPD performance and highlights the potential for further advancements in organic photodetection technologies.

**Figure 10:**
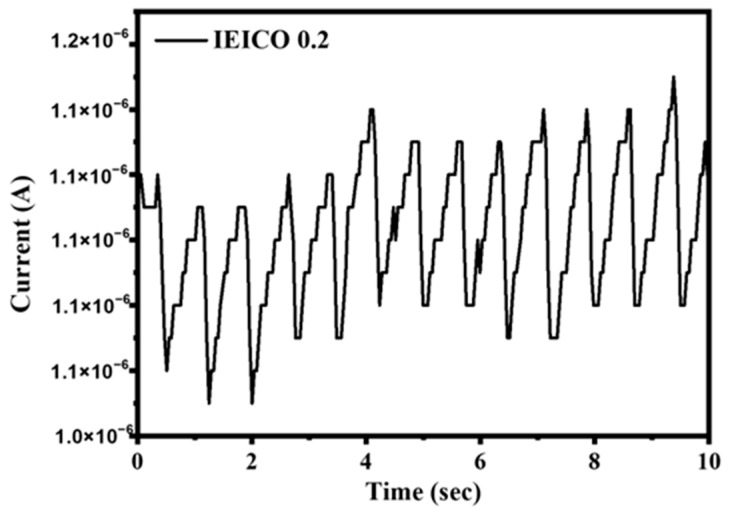
Direct current read-out of the device using 630 nm LEDs.

The heart rate measurement setup utilized in this study was established following methodologies outlined in a previous report, ensuring consistency and reliability in our experimental approach. Figure 10 depicts the heart rate of one of the authors at rest. The heart rate was calculated to be 78 beats per min^−1^ for the author at rest by dividing 60 s by the average interbeat interval. The outcomes derived from heart rate sensing at 630 nm showcased a remarkable level of precision and accuracy, underscoring the device’s reliable detection performance. The distinct and well-defined signal observed in our experiments serves to augment the device’s usability across a spectrum of applications, including but not limited to blood pressure monitoring, wherein precise and reliable measurements are paramount. Our meticulous measurements and analyses effectively highlight the device’s aptitude for heart rate sensing due to its expanded absorption spectra and consistently high performance. By judiciously selecting PM7 and IT-4F as the constituent materials, we have succeeded in realizing OPDs that not only meet but exceed expectations in terms of performance and functionality. This achievement underscores the efficacy of our approach and holds significant promise for the advancement of organic optoelectronic technologies in the realm of biomedical sensing and beyond.

## 5. Conclusions

In conclusion, our extensive research has led to the elucidation of a groundbreaking discovery: the phenomenon of vertical phase separation within the fabricated OPDs, which was substantiated by compelling AFM cross-section images and KPFM data. Furthermore, the integration of IEICO 0.2 into our device architecture has yielded unprecedented results that transcend all preconceived expectations, thus underscoring the transformative potential of our innovative approach. Notably, the OPD based on IEICO 0.2 showcases an exceptional dark current performance, registering a remarkably low value of 4.95 × 10^−10^ Acm^−2^ while simultaneously boasting an impressive conversion efficiency of 49%, a commendable responsivity value of 0.26 AW^−1^, and a remarkable detection ratio of 4.95 × 10^13^ Jones. Even when subjected to illumination at a wavelength of 780 nm, the OPD exhibits an LDR efficacy exceeding 100 dB, which is a testament to its unparalleled performance characteristics. Moreover, the observed cutoff frequency of 220 kHz represents a noteworthy achievement in terms of device operational capabilities. These remarkable achievements are primarily attributed to the inherent excellence of the binary device, a foundation further fortified by the strategic integration of IEICO-4F into its architecture, which is a move that effectively broadens the absorption spectrum and enhances the device’s overall performance. As a result, our findings not only contribute to the fundamental understanding of OPD operation but also pave the way for the realization of novel and efficient applications, particularly in the realm of heart-rate-sensing technology. This promising development holds immense potential for addressing existing challenges and driving innovation in the field of biomedical sensing, opening exciting new avenues for future research and technological advancements.

## Figures and Tables

**Table 1:** Comparison of work function values for pure materials.

Material	Work Function
PM7	−5.3647 eV
IT-4F	−5.6447 eV

**Table 2:** Performance characteristics of OPDs published in recent years.

Donor: Acceptor	Dark Current (A cm^−2^) (Bias)	R (A W^−1^) (Bias)	D* (Jones) (Bias)	Ref.
PM7:IT-4F: IEICO-4F	4.95 × 10^−10^ (−1 V)	0.26 (−1 V)	4.95 × 10^13^ (−1 V)	This work
PTB7-Th:COT-Oct	8.18 × 10^−9^ (−0.5 V)	0.24 (−0.5 V)	1.49 × 10^12^ (−0.5 V)	[20]
PCE10:YZ1	5.3 × 10^−11^ (0 V)	0.27 (0 V)	9.24 × 10^13^ (0 V)	[18]
PBDB-T: FM2	6.45 × 10^−9^ (−0.5 V)	0.455 (−0.5 V)	1.01 × 10^13^ (−0.5 V)	[19]
PM6:PY-IT	1.05 × 10^−9^ (−2 V)	0.44 (−2 V)	2.39 × 10^13^ (−2 V)	[1]
PM6:Y6	8.77 × 10^−10^ (−2 V)	0.516 (−2 V)	3.1 × 10^13^ (−2 V)	[10]
PffBT4T-2OD:PC_71_BM	1.2 × 10^−9^ (–2 V)	0.427 (–2 V)	2.9 × 10^13^ (–2 V)	[2]
PffBT4T-2OD:PC71BM:IEICO-4F	6.3 × 10^−11^ (0 V)	0.36 (0 V)	8 × 10^13^ (0 V)	[17]
D18:Y6	4.87 × 10^−8^ (−0.5 V)	1.21 (−0.5 V)	1.83 × 10^13^ (−0.5 V)	[33]
DTDCPB:C_70_	≈1 × 10^−9^ (−3 V)	0.30 (−3 V)	7.09 × 10^12^ (−3 V)	[11]
PM6:PDTTYM	3.88 × 10^−9^ (−2 V)	0.5 (0 V)	1.35 × 10^13^ (0 V)	[34]
PD004:PD-A2	≈2 × 10^−8^ (−4 V)	0.63 (−4 V)	6.6 × 10^12^ (−4 V)	[35]
PBDB-T:DO4F	≈1 × 10^−5^ (−2 V)	0.5 (0 V)	3.05 × 10^13^ (0 V)	[36]
PTB7Th:COTIC-4F:Y6	1.2 × 10^−9^ (−0.1 V)	0.41 (−0.1 V)	8.2 × 10^12^ (−0.1 V)	[37]
TQ-T:IEICO-4F	8.4 × 10^−3^ (−2 V)	0.09 (−2 V)	≈10^−10^ (−2 V)	[38]
PTB7-Th:PC_71_BM+ PTB7-Th: CO_i_8DFIC:PC_71_BM	≈1 × 10^−3^ (−1 V)	0.43 (−1 V)	8.8 × 10^10^ (−1 V)	[39]
PBDB-T:Y6	8.7 × 10^−8^ (−2 V)	0.5 (−0.5 V)	≈10^12^ (−0.5 V)	[40]
PTB7-Th:BFIC	≈10^−7^ (−3 V)	0.3 (0 V)	5.46 × 10^13^ (0 V)	[41]
PF:IT−4F	3.73 × 10^−10^ (−2 V)	0.37 (−2 V)	3.39 × 10^13^ (−2 V)	[14]
CuSCN:Y6	≈10^−9^ (−2 V)	0.12 (−2 V)	9.97 × 10^12^ (−0.5 V)	[42]
PTB7-Th:NTQ	1.5 × 10^−4^ (−2 V)	0.25 (−0.1 V)	3.72 × 10^12^ (−0.1 V)	[43]
P3HT:N2200	≈10^−5^ (−3 V)	0.07 (−2 V)	7 × 10^11^ (−2 V)	[44]

## Data Availability

The data presented in this study is available on request from the corresponding author.
